# Salutary Effects of Nutritional Ketosis for the Diseased Human Heart

**DOI:** 10.1007/s11883-025-01333-8

**Published:** 2025-08-28

**Authors:** Christopher D. Crabtree, Alex Buga, Yuchi Han, Orlando P. Simonetti, Jeff S. Volek

**Affiliations:** 1 Department of Radiology, The Ohio State University, Columbus, OH USA; 2 Department of Human Sciences, The Ohio State University, Columbus, OH USA; 3 Division of Cardiovascular Medicine, Department of Internal Medicine, The Ohio State University, Columbus, OH USA

**Keywords:** Ketones, Ketogenic, Ketone ester, Cardiovascular disease, Cardiovascular performance, Cardiac Function

## Abstract

**Purpose of Review:**

We provide an overview of cardiac metabolism, ketone physiology and terminology, methods of elevating ketones and their effect on cardiac function and disease. We discuss future research directions and speculate what ketogenic strategies may yield optimal effects on the heart and cardiovascular disease.

**Recent Findings:**

Nutritional ketosis acutely elevates cardiac function (cardiac output, myocardial perfusion, etc.) in healthy people and those with cardiovascular disease in a dose-dependent manner between circulating ketones and cardiac function. Despite therapeutic potential, long-term studies have not been performed. This acute effect is rapid, dose-dependent, and has been seen to be durable for up to 14 days following intervention onset.

**Summary:**

There are numerous methods to elicit ketogenesis and promote nutritional ketosis. There is growing evidence to suggest that higher ketone levels may offer greater cardiac benefits. It is pertinent to consider what ketone levels to target, and the best methods to safely and feasibly reach those targets over sustained periods of time.

## Introduction

Ketogenic interventions, including the ketogenic diet (KD) and administration of exogeneous ketones, aim to elevate circulating ketone bodies into a normal physiological range of nutritional ketosis (typically measured as beta-hydroxybutyrate in the range of 0.5 to 6 mM), whereby ketones function as a significant fuel source and signaling molecule. The KD has demonstrated efficacy in management of various chronic diseases with metabolically-impaired pathophysiology such as obesity, metabolic syndrome, prediabetes, and type 2 diabetes [1]. The use of ketones as a therapeutic agent for the diseased heart has recently gained newfound attention. Non-dietary ketogenic strategies including both ketone infusion and oral administration with various forms of exogenous ketones have been observed to acutely elevate cardiac function and blood flow proportional to plasma ketone levels [2, 3]. Each of the various methods of achieving nutritional or physiological ketosis has potential strengths and weaknesses/challenges, which should be considered by investigators, clinicians, and patients. This review provides a discussion of cardiac metabolism in health and disease, ketone physiology and terminology, methods of achieving elevations in ketones, and their effects on cardiac function and cardiovascular disease. We end with future research directions and speculation on which ketogenic strategies may yield optimal effects on the heart and cardiovascular disease.

## Cardiac Metabolism in Health and Disease

There is clear evidence of cardiometabolic distinction between healthy and diseased hearts. In healthy adults, the heart requires approximately 6 kg/day of ATP and has the highest oxygen consumption per unit weight of any organ in the body to meet its continuous high energy requirements [4, 5]. Due to variance in energy demands and substrate availability, the healthy heart is adaptable, utilizing a variety of metabolic substrates to maintain mechanical efficiency and function [6]. Oxidative phosphorylation provides the majority of ATP generation in the heart [7]. Healthy hearts (assuming a non-ketogenic state) primarily rely upon fatty acid oxidation (FAO), with glucose used as a secondary substrate to rapidly generate bulk energy [5, 6, 8]. Ketones are utilized by the heart in proportion to blood concentration [9] and since most people are not in a state of nutritional ketosis, it is generally a minor contributor to total energy demands. Additionally, lactate and amino acids can be used to generate ATP in relatively minor amounts under specific conditions, such as exercise.

The diseased or failing heart loses its characteristic metabolic flexibility and robust ATP generating capacity without a proportional loss of systemic blood flow demand. Although dependent on specific disease etiology and progression, the diseased heart has an approximate 30% reduction in ATP generation compared to healthy adults, which has been compared to “an engine running out of fuel [10]”. The failing heart undergoes structural remodeling as a maladaptive response to lack of blood flow, ensuring the heart can pump enough bulk blood at the cost of efficiency, function, and energetics [11]. This energy deprived state, compounded by a structurally remodeled heart, leads to metabolic remodeling [12], a reorientation of cardiac metabolism and substrate selection, reflective of the heart abandoning the now inefficient substrates toward selection of substrate with intact metabolic function.

The failing heart cannot efficiently utilize FAO, experiencing up to a 70% reduction in activity compared to healthy controls [13]; however, this metabolic impairment is not universally accepted [14], likely due to individual differences in disease progression and etiology [15]. Evidence indicates downregulation of enzymes involved in FAO [16], such as the rate limiting enzyme carnitine palmitoyltransferase I (CPT1) [4, 17] responsible for transport of long-chain fatty acids (FAs) into myocardial mitochondria [18]. The heart compensates by utilizing glucose, specifically glycolysis [19], in much greater proportion to overcome the continuous energy deficit [7, 13][20–22]. This fuel switch from FAs to glucose poses a significant problem for individuals who are also insulin resistant, which is common in patients with heart failure (HF) [23], characterized by impaired insulin mediated glucose uptake [24, 25]. Ketones freely diffuse into myocardial mitochondria for uptake [26, 27], bypassing both the impaired CPT1 transport [18] and insulin signaling systems [10, 18, 26, 28]. As an adaptive response, the heart utilizes ketones in greater proportion which generate ATP though oxidative phosphorylation [22]; however, ketones are not typically present in large enough supply due to the hypoketotic effect of modern carbohydrate-centric diets. Ketones cannot fully meet the cardiac ATP deficit and prevent further cascading disease progression without targeted interventions to achieve and maintain clinically beneficial ketone levels.

Ketones are readily utilized by the heart proportional to arterial concentration [29] without evidence of a myocardial threshold up to plasma levels approaching ~ 4 mM [30]. Ketones generate more ATP than FA per unit oxygen consumed [31]. While the failing heart shifts its metabolism to rely on glycolysis uncoupled from glucose oxidation [19], this generates increased protons, impairing contractility [32, 33] and decreasing cardiac efficiency [4]. Ketones on the other hand, may reduce vascular inflammation [34, 35] and ROS generation [36]. Ketones may also limit structural remodeling at early stages of cardiovascular disease progression in rats [37]. Ketone elevation and carbohydrate restriction can contribute to weight loss, improved body composition, feeling of well-being, glucose and insulin control [38], and reduce other biomarker risk factors for cardiovascular disease (CVD) and type 2 diabetes (T2D).

## Ketone Physiology

Ketone bodies are water soluble, short chain organic acid compounds derived from the partial breakdown of FAs. The three primary ketone bodies are acetoacetate (AcAc), beta-hydroxybutyrate (BHB), and acetone [39]. Ketone bodies are endogenously produced in mitochondria, primarily in hepatocytes (up to 300 g/day), through a process called ketogenesis. Long chain FA substrates delivered to the liver are transported from the cytosol via CPT1 on the hepatocyte outer mitochondrial membrane. Once inside the mitochondrial matrix, FAs are broken down to acetyl-CoA. Acetyl-CoA serves as the substrate for a series of metabolic reactions involving key ketogenic enzymes such as HMG-CoA synthase and HMG-CoA lyase to generate AcAc. HMG-CoA synthase catalyzes the rate-limiting condensation step, while HMG-CoA lyase cleaves HMG-CoA to form acetoacetate. AcAc can then be converted into BHB by beta-hydroxybutyrate dehydrogenase (BDH).

Ketogenesis is regulated primarily by plasma insulin and glucagon concentrations. Insulin potently downregulates lipolysis and promotes lipogenesis within the physiological ranges observed in humans. Glucagon activates hormone sensitive lipase, promoting lipolysis and releasing FAs to fuel ketogenesis. Ketone and glucose metabolism resemble a see-saw, where in periods of carbohydrate restriction (e.g., KD) or abstinence (e.g., prolonged fasting) and low circulating supply of insulin, lipolysis and ketogenesis are accelerated, manifesting in a markedly increased availability of ketones, whereas glucose abundance (e.g., modern diets) has the opposing effect, and suppresses ketogenesis.

Ketones are readily utilized by most extrahepatic tissues although at different organ-specific utilization rates [40], with the brain and heart being two primary consumers. Ketone bodies are also important pleiotropic signaling metabolites that inhibit histone deacetylases (HDAC) and modulate G-protein coupled receptors [26, 41]. Additionally, ketones, through epigenetic signaling, reduce inflammation and oxidative stress [26, 42], particularly in specific chronic diseases featuring dysfunctional metabolism, such as heart failure [43]. Whether in response to prolonged starvation or a KD, there is a highly coordinated set of genetic, physiological, and metabolic adjustments that occur to ensure normal metabolic functioning. We have described this process as “keto-adaptation”, which is associated with decreased reliance on glucose oxidation and accelerated rates of lipolysis and ketogenesis [44], ketone uptake and oxidation [44], weight loss in the context of individuals with excess adiposity [45, 46], improved glycemic and lipid/lipoprotein management [47], and normalization of insulin sensitivity [48]. There are many other physiological changes associated with keto-adaptation that are beyond the scope of this review, but a major relevant question currently under investigation by multiple research groups is how the heart may respond over time in response to different ketogenic interventions.

## Defining Ketosis

**Fig. 1:**
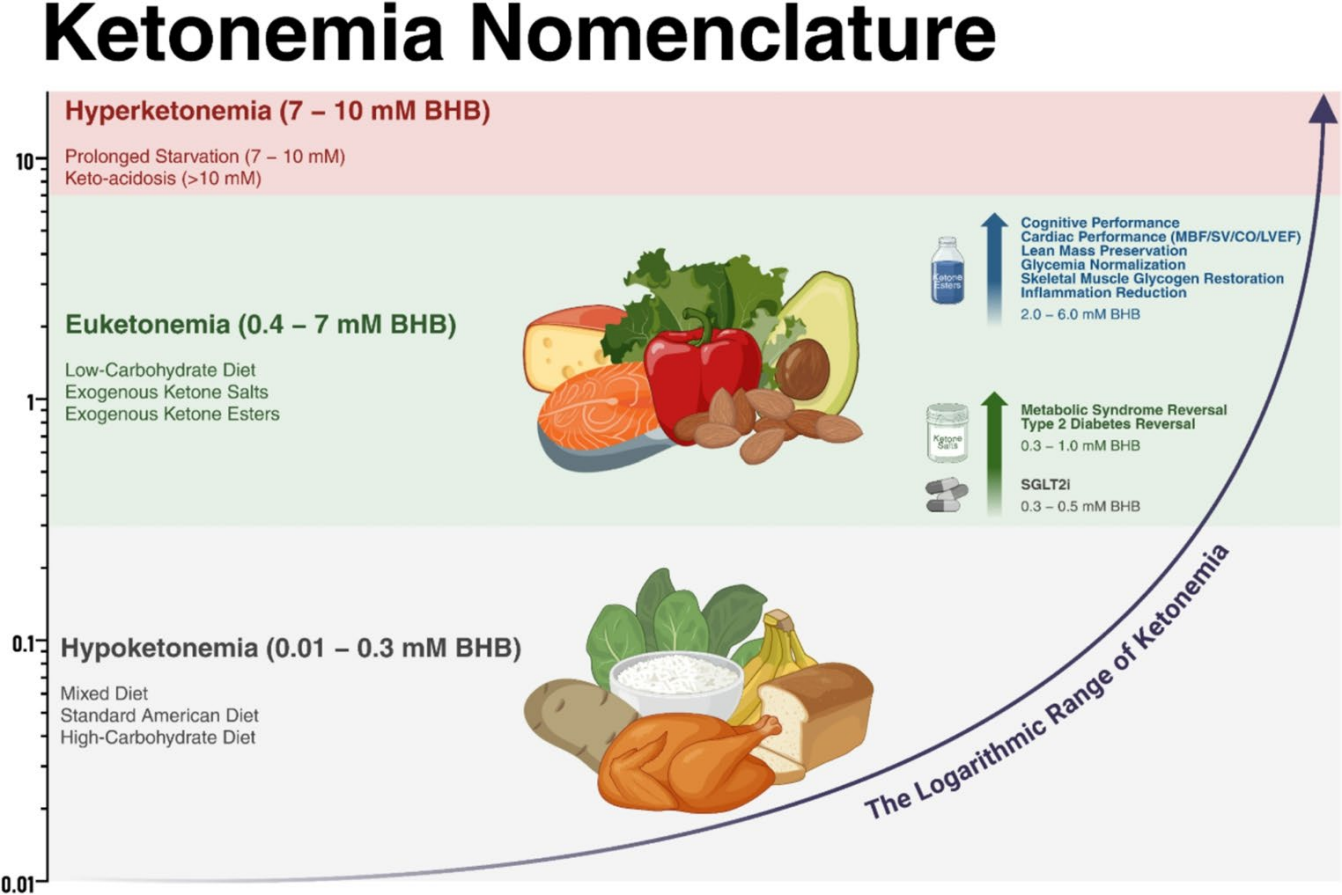
Circulating ketones span over four orders of magnitude [< 0.01 to > 10 mM], each representing a distinct metabolic physiology with corresponding adaptations. Contemporary evidence suggests higher ketone levels (2–6 mM BHB) may be optimal for comprehensive clinical benefit beyond lower ketone levels conferred from carbohydrate abstinence or acute exogenous ketone supplementation alone

Ketogenesis is a highly conserved pathway that had a critical role in providing a lipid-based source of fuel for the brain and other tissues during periods of food scarcity. Remarkably, circulating ketones can span over 4 orders of homeostatic magnitude in humans (< 0.01 to > 10 mM) (Fig. 1). Most people who consume a diet that contains more than 150 g/day of carbohydrate have circulating ketones, often measured as BHB, less than 0.3 mM after an overnight fast. Thus, we arbitrarily define nutritional ketosis as starting above 0.3 mM and extending to 4–6 mM, the upper end being commonly observed in people during prolonged starvation. Physiological or nutritional ketosis is distinct from the much higher levels associated with the pathological state of keto-acidosis, usually due to insufficient insulin resulting in proton overproduction and overwhelmed buffering capacity [49].

Since nutritional ketosis is an essential and normal human metabolic state spanning the lifespan, we proposed the term “euketonemia” to describe ketogenic interventions that elevate ketones into the beneficial range of nutritional ketosis (BHB = ~ 0.5 to 5–6 mM) [50]. Euketonemia is distinguished from pathological ketone levels observed in keto-acidosis or “hyperketonemia”. If achieving euketonemia is a goal in the context of heart disease, it is critical to determine the optimal level of nutritional ketosis needed to elicit clinical benefit. Equally important is identifying and implementing the preferred method for achieving nutritional ketosis in order to maximize long-term adherence and benefit.

## What Are Optimal Ketone Levels?

Remarkable benefit has been consistently demonstrated in reversing metabolic syndrome and type 2 diabetes with a well-formulated ketogenic diet (WFKD) that achieves blood R-BHB concentrations at the lower end of nutritional ketosis (~ 0.3 to 1 mM) [51, 52]. This is similar to the levels achieved with sodium-glucose cotransporter-2 inhibitors (SGLT2i), medications that are associated with long-term cardiac benefits [53, 54]. It is tempting to speculate that targeting higher ketone levels may be necessary to elicit optimal clinical outcomes for some indications, including reduced cardiac function, while also capturing a subset of non-responders; however, definitive data has not yet been generated to support such a conclusion. Although this “more is better” philosophy regarding ketone concentrations may seem overly simplistic and naive, it is grounded in scientific evidence that should be rigorously debated and tested.

Several lines of evidence support the hypothesis that the upper range of nutritional ketosis (~ 2–6 mM) may be necessary to elicit optimal therapeutic benefits. This is based in part on established dose-response associations between ketone concentrations across a broad range (0.1 to > 10 mM) governing both brain [55] and heart [30] ketone uptake and oxidation. Functionally, this translates into ketone-dependent improvement in cognitive [56] and cardiac [3, 57] performance, including cardiac output (CO) and myocardial perfusion. Ketone concentration exhibits a dose-response relationship with a wide range pf physiological and signaling effects such as cardiac function in healthy and diseased individuals [3, 57], skeletal muscle glycogen restoration [58], inhibition of histone deacetylase (HDAC) activity [59], and inhibition of nod-like receptor protein 3 (NLRP3) inflammasome activation [42]. The potential clinical benefit of achieving higher ketones is further buttressed by clinical observations and case studies that demonstrate a positive correlation between ketones and retention of lean mass during weight loss in people with type 2 diabetes [60], seizure control in children with epilepsy [61], decreased metastases and increased survival in animal models [62] and quality of life in people with various types of cancer [63].

Not all studies support a ketone dose-response effect, but this could be due in part to the use of traditional single morning measurement of total ketone exposure that fails to capture diurnal/nocturnal variability. Moreover, this does not rule out potential threshold effects (i.e., reaching a certain ketone level to elicit a maximal effect) or nonlinear associations. Additionally, many acute exogenous ketone supplementation studies lack the temporal resolution to investigate this relationship.

Currently, there is biological and physiological evidence to target the higher end of euketonemia for optimal clinical outcomes, especially in heart disease [3]. However, long-term clinical outcome data is sparse. Future clinical studies should prioritize frequent measurement of ketones with repeated ketone measurements throughout the day. It is important to associate clinical outcomes with not just the presence of nutritional ketosis, but specific level of ketones to determine optimal target ranges.

## Methods of Achieving Nutritional Ketosis

There are multiple methods of achieving nutritional ketosis, most notably the (KD) and exogenous ketone formulations, which are discussed in detail here. Additional methods include prolonged fasting, high volume exercise, infusion, and SGLT2i medications.

### Ketogenic Diet

The KD is one of the most studied dietary patterns with overwhelming evidence supporting safety and efficacy across multiple diseases. The comprehensive details of formulating and implementing a KD are beyond the scope of this review and detailed elsewhere [1]. Briefly, from a macronutrient perspective, it is very low in carbohydrate, moderate in protein, with varying levels of fat depending on caloric intake. KDs can be formulated in a variety of ways but are ideally comprised of whole foods that include a variety of non-starchy vegetables, low-sugar fruits, fatty meats, full-fat dairy, nuts/seeds, butter, eggs, and other low/zero-carbohydrate foods such that total carbohydrate is less than 50 g/day. Saturated and monounsaturated fats from natural sources are emphasized with attention to avoid excessive intake of polyunsaturated fat. Special attention is given to higher sodium and potassium intake as the diet has a natriuretic effect. These principles and others [1] comprise what we have termed a *well-formulated ketogenic diet* (WFKD) to distinguish it from variants such as the more restrictive historical use of KDs in the management of pediatric intractable seizures [64, 65].

While it is possible for some people to achieve ketone concentrations at the higher end of euketonemia (3–6 mM) levels with a strictly designed KD alone, most free-living people consuming a sustainable, sapid, whole food-based WFKD demonstrate average blood R-BHB levels in the 0.3 to 1 mM range with tremendous interindividual variability [66, 67]. Even under tightly controlled metabolic ward conditions, a group of lean healthy male athletes fed a eucaloric KD consisting of meat and fat with no vegetables (85% of energy as fat and 15% protein) had mean fasting BHB values of 2.2 mM across 4 weeks of close monitoring [68].

To achieve higher ketones, some people may be encouraged to adopt a combination of secondary methods of ketone stimulation: excessive exercise, prolonged fasting, or unnecessary restriction of nutrient dense foods – all of which have potential adverse effects. Not to mention such practices may be impractical or unsafe for many people, especially for patients with heart disease, and those who are frail or critically ill.

The temptation to restrict otherwise important foods on a WFKD to elevate ketones may compromise enjoyment and diet compliance while increasing risk of essential nutrient deficiencies. For example, to achieve higher ketones, non-starchy vegetables and low-carbohydrate fruit (sources of essential minerals) may be reduced or eliminated and/or protein may be restricted to a critical threshold that promotes unfavorable lean mass losses. Any ketone-enhancing benefits in these cases would not offset the harm. Prolonged fasting beyond a few days is not advised as it can have cumulative adverse effects on mineral balance, nitrogen loss and muscle mass among other undesirable effects.

The epiphenomena of many standard-of-care medical treatments (e.g., exogenous insulin or insulinotropic drugs, chemo or radiation therapy, steroids, statins, psychiatric medications, etc.) are inherently anti-ketogenic, creating further barriers to achieving nutritional ketosis with a KD alone. An exception is the class of SGLT2 inhibitor drugs increasingly being used in individuals with heart disease that reverse hypoketonemia into the low range of nutritional ketosis (0.3 to 0.7 mM) [69].

A single arm KD intervention found improvements in various cardiometabolic related markers including body weight, blood pressure, low-density lipoprotein cholesterol, high-density lipoprotein cholesterol, and total cholesterol in healthy adults with mildly elevated LDL-C [70]. Numerous other studies have shown similar improvements in risk factors for CVD and metabolic syndrome more broadly, while on a KD [45, 52, 71, 72]. The lack of KD research in HF can be attributed to multiple factors. First, KD fall outside traditional mainstream dietary practices advocated for in the Dietary Guidelines for Americans and professional organizations such as the American Heart Association. The American Diabetes Association, however, has recently updated their guidelines to include low-carbohydrate dietary patterns as an option. Thus, the limited long-term dietary trials that have investigated hard end points have studied primarily low-fat dietary patterns. Second, high-quality dietary interventions are difficult to implement and expensive to conduct, requiring a multi-disciplinary team to manage dietary interaction with traditional longitudinal clinical markers. KD require careful attention to detail to properly educate and support participants, which may be difficult for some who have been taught traditional nutritional views, such as the “food pyramid” built on a base of carbohydrates. Although KD is by far the most well-studied ketogenic approach, the advent of exogenous ketone products has ushered in a new method of achieving nutritional ketosis.

### Exogenous Ketones

Consumption of exogenous ketones and ketogenic-promoting precursors, with or without a WFKD, is an alternative strategy for eliciting nutritional ketosis. Exogenous ketones do not reduce insulin nor augment fatty acid oxidation like a WFKD [66], and thus might better be viewed as an amplifying agent rather than as a replacement for nutritional ketosis elicited by carbohydrate restriction. Exogenous ketones aim to increase circulating ketones and contain calories, qualifying as foodstuff. Most commercially available products have a generally recognized as safe (GRAS) status and are usually sold as a liquid beverage or powder. Exogenous ketones consist of varying compositions such as direct forms of BHB or AcAc as an acid or salt, or joined with other ketone-promoting components by ester bonds. Alternatively, exogenous ketones may instead contain ketogenic precursors that metabolize to ketones in the liver (i.e., medium chain fatty acids), or ketone precursors that metabolize to BHB via non-classical metabolic pathways. Current formulations are plagued by objectionable odor and taste, that adversely affect palatability and tolerance, although this is improving with flavor innovation in new products.

Exogenous ketone formulations have differing kinetics and likely physiological responses. However, there have been only limited head-to-head comparisons of various formulations. Exogenous ketones have different acidic/alkali signatures based on their formulation, with salts being alkalinizing and esters being slightly acidifying. The acid load with ketone esters is mild, even with relatively frequent high use, and within the body’s buffering capacity such that bicarbonate, carbon dioxide, and anion gap remain within normal limits given reasonably normal kidney function.

**Fig. 2:**
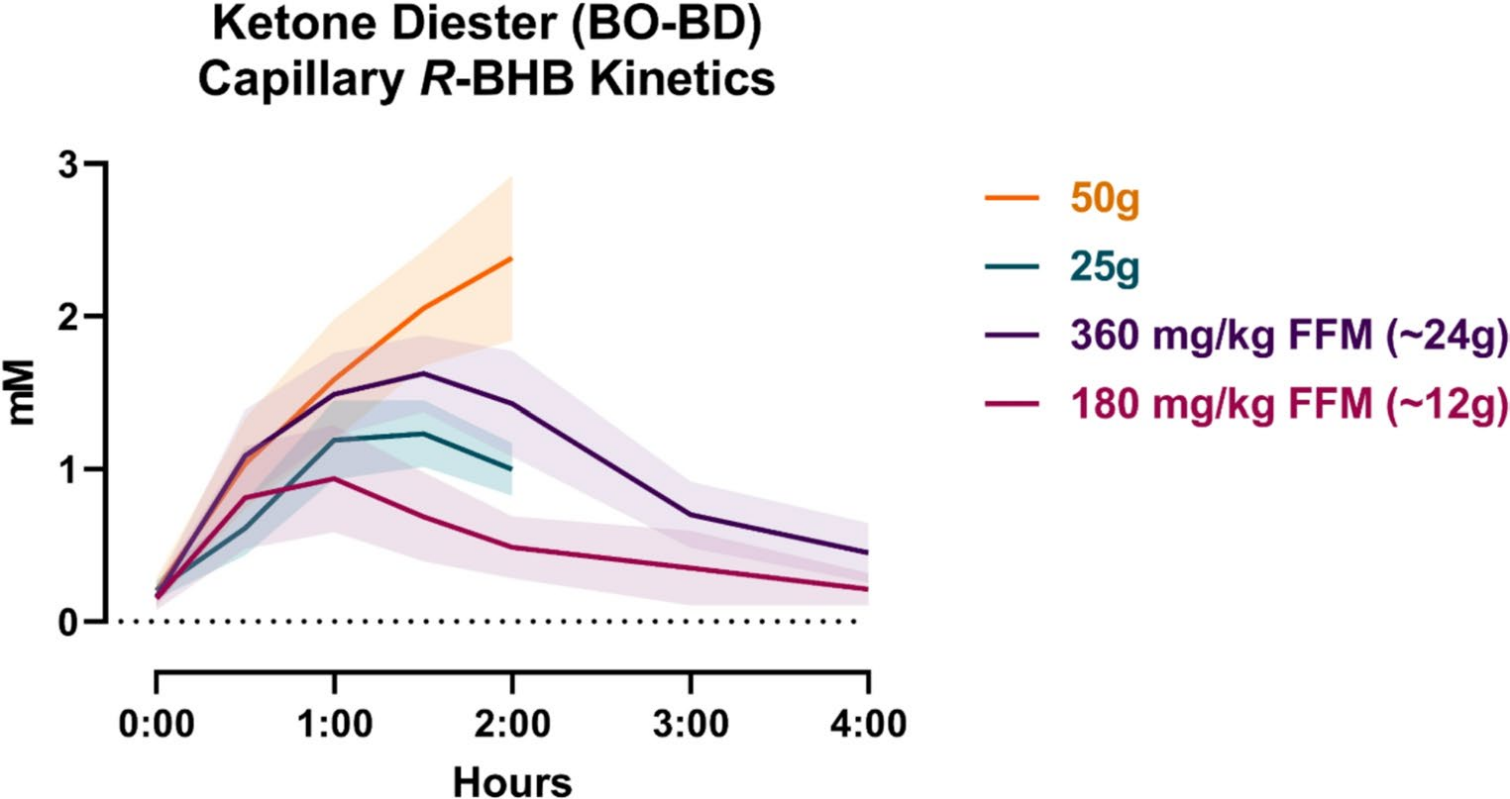
Capillary *R*-BHB Pharmacokinetics. A comparison across four separate studies, all in healthy adult cohorts, of BO-BD R-BHB plasma kinetics between two flat-doses (25g and 50g KE) compared to relative-doses (180 mg/kg and 360 mg/kg, both relative to fat free mass (FFM)). Data presented as mean ± 95% confidence interval clouds. Data from [74] and unpublished data from ongoing laboratory studies

We have substantial experience exploring metabolism of C8 Diester in humans, which is a pro-ketone that consists of bis-octanoyl (R)−1,3-butanediol. Ingestion of C8 Diester is well tolerated with single doses as high as 50 g (Fig. 2**)** [73]. We have shown that an earlier version of this compound C6 Diester (bis-hexanoyl (R)−1,3-butanediol) rapidly elevates plasma ketones and cardiac function in a dose dependent manner for a period of hours [57, 74].

There are several reasons the C8 Diester may be a preferred candidate as a foundational ketone-promoting compound. The ketogenic precursor components of the C8 Diester are octanoic acid (i.e., caprylic acid) and (R)−1,3-butanediol (BDO) in a ratio of ~ 3:1 by molecular weight. The C8 Diester is rapidly metabolized by esterase enzymes in the small intestines or in plasma to release their constituent molecules, which accelerates endogenous ketogenic pathways through hepatic conversion to ketone bodies.

Octanoic acid is a ketogenic-promoting medium chain saturated FA that is not appreciably stored in adipose triglycerides, cell, or organelle membranes. It bypasses traditional regulation required for absorption and oxidation of long-chain fatty acids (e.g., lymphatic absorption and CPT1-mediated transport across the mitochondrial membrane). Therefore, octanoic acid is a hierarchically preferred substrate for beta-oxidation. When adequately supplied, it is absorbed into the portal vein, transported to the liver, and diverted toward the traditional hepatic ketogenesis pathway. In isolated form, octanoic acid increases both BHB and AcAc to a greater extent than capric acid (C10) and lauric acid (C12) [75]. The ketogenic effect of octanoic acid is limited when consumed with a carbohydrate-containing meal [76]. This attenuation likely reflects elevated insulin levels that suppress lipolysis and beta-oxidation, thereby constraining the acetyl-CoA pool necessary for ketogenesis. Conversely, carbohydrate abstinence during a WFKD facilitates a larger acetyl-CoA pool from accelerated lipolysis and beta-oxidation, enabling further potentiation of ketogenesis when supplemented with octanoic acid.

The medium and long-chain FA substrates for the normal hepatic ketogenic pathway produce both ketone bodies BHB and AcAc; this is an important feature in mediating downstream benefits attributed to accelerated ketone metabolism. For example, multiple human studies using the forearm arterial-venous difference model have demonstrated that AcAc uptake in muscle is increased as keto-adaption proceeds with a corresponding net release of BHB back into the venous circulation [77–79]. This skeletal muscle ketone conversion process contributes to consistently higher concentrations of plasma BHB than AcAc. The reaction is catalyzed by BHB dehydrogenase, which results in the conversion of a molecule of NADH to NAD^+^. NAD^+^ is well known for its role in redox reactions, but more recently it has emerged as a key signaling molecule controlling a myriad of processes from energy metabolism to cell survival [80]. Declining levels with age are associated with impaired metabolism and increased disease susceptibility. If and how exogenous ketones impact NAD^+^ has not been explored but is likely an important feature to consider.

The other component of the C8 Diester, (R)−1,3-butanediol (BDO), is converted to ketone bodies in the liver through a separate pathway that involves alcohol and aldehyde dehydrogenase. A BDO dose of ~ 12 g is rapidly absorbed and incrementally increases peak R-BHB by ~ 1.0 mM 1–2 h after ingestion. Doubling and tripling the BDO dose (~ 25 and 35 g) results in a diminishing return in terms of peak R-BHB (~ 1.4 and 1.8 mM, respectively), but the incremental area under the curve over 4-hours is more than doubled [81] (*unpublished data from our lab*). Typically, R-BHB begins to gradually decline after 2–3 h but levels remain above baseline out to 5-hours. Based on unpublished data, we have observed that plasma AcAc is also elevated after BDO ingestion, but to a smaller extent than R-BHB.

The incremental area-under-the-curve (iAUC) and maximum concentration (Cmax) kinetic responses to ingestion of C8 Diester in humans are higher in people habitually adapted to a KD [74], supporting a dual approach to achieving therapeutic nutritional ketosis. A KD upregulates the ketogenic synthetic machinery at rest and post-exercise, by increasing constitutive gene expression of 3-hydroxy-3-methylglutaryl-CoA synthase 2 (HMGCS2), the rate-limiting enzyme of ketogenesis [82]. In the presence of increased FA substrates provided by C8 ingestion, ketogenic efficiency is accelerated in part because the ketogenic pathway is already primed.

Augmenting exogenous ketone kinetics may also be the case with exogenous ketone-salts that contain pre-formed BHB but not AcAc [83]. This may be due to non-hepatic keto-adaptations, such as significantly reduced renal excretion of ketones [84], such that in the keto-adapted state more of the BHB ingested is conserved (i.e., less loss in urine). Outside of the pioneering work by George Cahill and colleagues circa 1960s and 1970s documenting the human ketogenic response to prolonged starvation [85], there is a dearth of detailed research on the temporal adaptations in renal handling of ketones in response to a WFKD.

### Ketogenic Optimization: Emerging Strategies

Another approach worth considering in future research and implementation is layering separate exogenous ketone compounds such as BHB-salts, BHB free acids, or utilizing BHB enantiomeric ratioing, to achieve optimal pH- and metabolic-dependent effects for specific disorders/diseases, physiological targets, and operational contexts. BHB is a chiral molecule with the primary enantiomer of R-BHB (or D-BHB) produced during endogenous ketogenesis. R-BHB is readily utilized for ATP production, matching the chiral specificity of BHB dehydrogenase [86]. For this reason, traditional enzymatic assays to quantify ketones, including handheld point of care devices, only measure R-BHB. The other enantiomer, S-BHB (or L-BHB), is less well studied, especially in humans. Racemic mixtures of R-BHB and S-BHB (i.e., 50:50) are common in commercially available ketone supplements, but product purity is not readily known and can vary (e.g., we have tested products with non-racemic ratios of R- and S- enantiomers) [66].

Limited information is available on the presence of S-BHB in plasma. In rats, mean values of serum R-BHB and S-BHB were 106.2 and 3.9 µM, respectively [87]. Tissue levels of S-BHB in rats demonstrate widespread distribution with the highest enrichment observed in the heart [87, 88]. Although the source of S-BHB remains controversial, these data highlight the notion that S-BHB is a ‘natural’ minor ketone body.

The biological origin and functional significance of S-BHB remains speculative, but new research highlights powerful cardiac and hemodynamic effects after an infusion of S-BHB elevated plasma ketones higher and longer than R-BHB, which in turn led to a greater elevation in cardiac function [89]. S-BHB is generally not considered an important oxidative fuel, but it has been shown to be a preferred ketone substrate for sterol and fatty acid synthesis in kidney, liver and central nervous system [90], regulator of cardiac glucose metabolism [87], and scavenger of reactive oxygen species [91]. It is also likely that S-BHB retains putative signaling functions attributed to BHB such as HDAC or NLRP3 inflammasome inhibition, which do not appear to demonstrate chiral specificity [41].

We have recently developed a sensitive and accurate liquid chromatography with tandem mass-spectrometry (UPLC-MS/MS) assay method to quantify S-BHB and made several observations [66, 74, 83]: (1) S-BHB is normally present in human plasma, albeit at much lower concentrations than R-BHB (i.e., usually < 5% of total BHB), (2) plasma S-BHB increases > 5-fold within a few weeks of consuming a KD and (3) the kinetics of plasma S-BHB are different than R-BHB, displaying longer concentration elevation, likely reflecting delayed clearance. Thus, one rationale for potentially using both enantiomers of BHB is that R-BHB will be absorbed more quickly for an early rise in plasma BHB while S-BHB has a longer half-life that would promote a more sustained and balanced elevation of both plasma R-BHB and S-BHB.

**Fig. 3:**
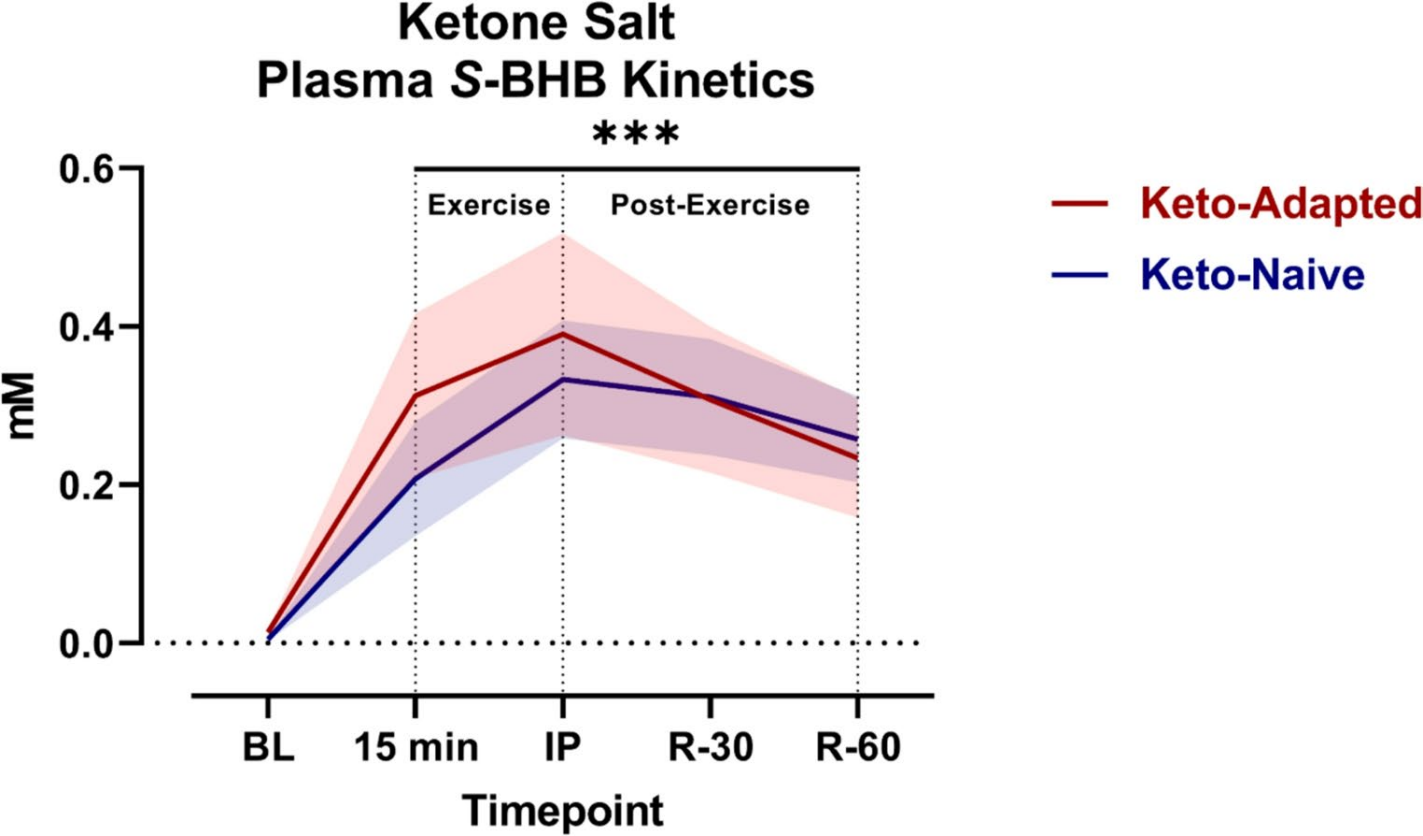
Venous ***S***-BHB Pharmacokinetics. S-BHB responses while fasting at baseline (BL) followed by ingestion of a non-racemic ketone salt at rest (15min), immediately post exercise (IP), and during exercise recovery (R-) sampled 30 (R-30) and 40 (R-40) minutes post-exercise. Data presented as mean ± 95% confidence interval clouds. Data from [84]

Relatively small doses of S-BHB are rapidly absorbed and proportionally increase to a greater extent than R-BHB, but with high variability across individuals. For example, ingestion of a non-racemic ketone salt product that contained ~ 2 g of S-BHB exhibited a profound elevation in S-BHB after just 15-min that for some subjects exceeded 0.5 mM and in others barely increased at all [83] (Fig. 3). In that experiment, subjects performed a staged high-intensity cycle ergometer time to exhaustion test 15-min after the supplement was co-ingested with caffeine, which was associated with a significant 8–10% performance improvement.

In summary, exogenous ketone and pro-ketones are a safe and viable method of achieving transient nutritional ketosis that could be used to enhance cardiac function with or without altering other dietary components. Frequent use and relatively high doses would be necessary to achieve sustained euketonemia over weeks and months if not combined with some level of carbohydrate restriction. Alternatively, a WFKD combined with exogenous ketones could be additive or synergistic, but this has not been examined in the context of cardiac function and cardiovascular disease clinical outcomes.

## SGLT2i, Ketosis, and Cardiovascular Responses

The current interest in ketones as a therapeutic for improving depressed cardiac function is partially attributed to the success of the SGLT2i. SGLT2i have become a mainstream clinical therapeutic option, currently included by the American Heart Association for standard of care treatment of heart failure [92], and the American Diabetes Association for lowering glucose [93] and reducing cardiovascular [94] and renal risk [95]. The inhibition of SGLT2 allows for greater urinary excretion of glucose, thus decreasing blood glucose levels and managing insulin response [96]. Before long-term outcome data from SGLT2i trials were available, therapies lowering blood glucose did not always reduce cardiovascular events, hospitalizations, or death [97, 98], despite the well evidenced and accepted knowledge that a major complication of chronic T2D was increased CVD [99, 100].

In one of the first studies to show a cardiovascular benefit of SGLT2i, 7020 patients received either a low or high dose of SGLT2i (empagliflozin) or placebo once daily in addition to standard care in T2D patients at high cardiovascular risk [53]. The group receiving empagliflozin demonstrated significant reductions in cardiovascular death (−38%), hospitalization for HF (−35%), and all-cause mortality (−32%) compared to the placebo group [53, 54]. These findings have been reproduced numerous times in various studies with broader inclusion criteria [101], different drug brands [102, 103], in non-diabetic rats [104], and non-diabetic humans [105–108]. Multiple meta-analyses have been conducted and found persistent improvements in outcomes relating to CVD in T2D patients [109–111]. However, improved clinical outcomes have not manifested in imaging measures of cardiac function determined by a meta-analysis of 11 relevant trials comparing SGLT2i to placebo [112].

Blood ketone concentrations are persistently elevated with SGLT2i use [113, 114]. While this elevation is modest (approximately 0.3–0.5mM) relative to other ketogenic methods, it lasts throughout SGLT2 inhibition and occurs regardless of metabolic health status [113, 114]. Interestingly, plasma ketone levels have been observed to further increase following extended dosing (4wk) compared to acute dosing [113]. Ketone elevation is not fully explained by changes to concentrations of plasma fatty acids [114], but is accompanied by increased ketogenesis, lipolysis, lipid oxidation, and a lower insulin to glucagon ratio [113], all favoring endogenous ketone production.

## Cardiovascular Response To Intravenous and Oral Exogenous Ketones

There has been a recent research thrust examining the acute cardiovascular response to infusing and consuming exogenous ketones. While this work is motivated by future translational impact for patients, these studies have been implemented in both healthy and diseased populations.

### Acute Intravenous Ketone Administration

Initial acute studies explored the impact of ketones on cardiac function using intravenous sodium (Na) −3-OHB administration. Gormsen et al. conducted a randomized crossover trial in eight healthy elderly participants, employing ketone infusions combined with a hyperinsulinemic-euglycemic clamp to control endogenous metabolism [2]. At peak [BHB] (3.78 mM) after 6.5 h, heart rate rose 25% and global myocardial blood flow (MBF) surged 75%. Myocardial glucose uptake decreased by 50% despite enhanced perfusion, indicating preferential ketone utilization when available.

Subsequent research examined the effects of ketone infusions versus placebo in individuals with HF, revealing dose-dependent cardiovascular improvements [3]. At 3.3 mM BHB, CO (+ 42%), stroke volume (SV) (+ 27%), and left-ventricular ejection fraction (LVEF) (+ 23%) increased accompanied by reduced systemic (−30%) and pulmonary vascular resistance (−21%). Nielsen et al. extended these findings to pulmonary hypertension patients [115], demonstrating 27% higher CO and 18% lower PVR at 3.4 mM BHB, with consistent effects across clinical subgroups.

### Acute Exogenous Ketone Ingestion (Ketone Esters)

Recent work has investigated the cardiovascular response to ketone elevation via KE consumption rather than ketone infusion, trading experimental metabolic control with improved practically toward free-living contexts. Ketone ester administration in 20 healthy adults achieved an average [BHB] of 3.2 during TTE imaging; HR (+ 15%), SV (+ 8%), LVEF (+ 5%), and GLS increased following infusion [116]. Of note, GLS increased independently of heart rate while SVR decreased − 16%, pointing to a potential inotropic effect. Patients (*n* = 13) suffering from cardiogenic shock (CS) demonstrated improvement in CO, LVEF, cardiac power output, and mixed venous saturation following ketone supplementation compared to placebo [117]. Compared to a water control, KE elicited a similar response to prior studies, elevating ketones, CO, HR, and SV, throughout a 60-minute time course in healthy adults [118]. In a separate trial, KE ingestion improved CO, MBF, SV, HR, and myocardial strain over the course of 120 min compared to an energy and volume matched fat based placebo drink in adults [57]. In both trials, the cardiovascular response began rapidly, with CO increasing within 15–30 min of supplementation.

The cardiovascular improvements across trials are clinically meaningful and the effects are similar to other currently prescribed inotropic agents [117], although the degree of clinical significance has not been described [30]. Compared to other inotropic agents such as dobutamine, ketones improve cardiac output, perfusion, oxygen delivery, and increase oxidative metabolism proportional to ketone elevation without impairing myocardial external efficiency (MEE) [3, 119]. Improvements in myocardial contractility [3, 116, 117] occurred independently of preload and HR [116], as well as health status. Ketones acting as an inotropic agent have been observed elsewhere in rodent models [119] and in COVID patients [120] exposed to ketones.

There are important distinctions to draw between SGLT2i and acute studies of exogeneous ketone administration. First, the level of blood ketones reached from exogenous ketosis (~ 3mM) is an order of magnitude higher than the modest levels conferred via SGLT2i (~ 0.3–0.5mM). The dose response relationship between cardiac function and [BHB] may explain why SGLT2i have not resulted in provocative changes to cardiac function or structure when examined with cardiac imaging [112], as one may expect given improvements in longitudinal outcomes.

While these acute studies do not provide evidence that ketones are responsible for the outcomes seen in the SGLT2i trials, they do showcase the profound effects that ketones have on cardiac function, and the therapeutic potential they may offer. These agents only elicit a response for a period of hours. To achieve maximal ketone benefit, plasma ketones have to remain elevated throughout not just the full day, but presumably in totality throughout the intervention period. Recent work has begun investigating the therapeutic potential of ketogenic agents when consumed daily but there remain many considerations and protocol optimizations required before large scale implementation.

### Chronic (14-day) Exogenous Ketone Ingestion (Ketone Esters)

Two studies thus far have been completed with continuous exogenous ketosis ingestion via two-week intervention regimens in heart failure with preserved ejection fraction (HFpEF) and heart failure with reduced ejection fraction (HFrEF) + T2DM populations [121–123]. A crossover study involving 24 HFrEF patients compared 14-day interventions of KE and placebo beverages, with a two-week washout period in between supplement crossover [121]. Testing occurred after each intervention without baseline visits. KE consumption improved resting cardiac output (+ 5%) and reduced pulmonary capillary wedge pressure (−20%) at trough ketone levels, with effects amplifying after acute supplementation. During exercise, these improvements persisted without affecting overall performance. A similar study in HFpEF patients with T2DM (*n* = 24) showed comparable results, with additional improvements in SV and pressure-flow relationship during peak exercise [123]. Both studies demonstrated sustained cardiovascular benefits over 14 days, suggesting limited tachyphylaxis. However, the absence of true baseline visits leaves this potential limitation unresolved.

## Nutritional Ketosis for the Diseased Heart: Future Directions

There are numerous special considerations required for continuous exogenous ketogenic therapeutic interventions. While studies investigating exogenous ketosis response to KE co-ingested with a meal is limited, food consumption (while somewhat dependent on macronutrient composition) does not neutralize ketone response [74]. However, it is unknown how the complexities of post-prandial metabolism interact with exogenous ketone elevation in the daily free-living context. There is a large degree of intersubject variability in plasma ketone response following exogenous administration, making it difficult or impossible to design a generalized protocol. Frequent measurement of ketones is therefore needed to understand how the individual is responding and adjusting the diet and/or exogenous ketones to achieve a target ketone range.

It must be profoundly stated that exogenous ketones do not mimic the broad-spectrum effects of a WFKD, which are only partially attributed to the development of nutritional ketosis. For example, an essential feature of keto-adaptation is the dietary carbohydrate restriction-mediated decrease in insulin secretion that in turn promotes enhanced fatty acid oxidation and ketogenesis. Exogenous ketones without carbohydrate restriction do not reduce insulin. Thus, optimal use of exogenous ketones may be as an adjunct to a WFKD, supplying an auxiliary source of ketones, rather than as a WFKD replacement.

Exogenous ketones alone are unlikely to be sufficient to elicit sustained optimal levels of ketosis; however, a WFKD combined with exogenous ketones could be additive or synergistic. Thus, we believe that to achieve optimal therapeutic nutritional ketosis, additional ketone-promoting strategies beyond a WFKD may be required. Although a hypothesis, we believe adjunctive use of exogenous ketones may be a compelling strategy to complement a WFKD for optimal therapeutic and functional outcomes without deleterious effects. Such an approach may facilitate highly personalized and dynamic treatment plans that address individual circumstances and a range of desired ketone levels (0.5 up to 5–6 mM) that target underlying pathologies, manage side effects, enhance physical and mental performance, and optimize clinical outcomes and quality of life.

## Conclusion

Ketogenic interventions demonstrate significant potential as metabolic therapeutics for diverse clinical populations by providing efficient myocardial substrates. A key strength lies in the versatility of ketosis-inducing methods, including dietary modifications, exogenous infusions, supplementation, and perhaps a combination of all three —enabling tailored approaches for individual preferences and clinical contexts. Acute studies consistently reveal robust cardioprotective effects, with rapid left ventricular functional improvements lasting several hours, directly correlating with plasma ketone concentrations. Preliminary investigations suggest that acute benefits persist during prolonged daily administration, supporting feasibility for long-term use. It is likely that maintaining sustained nutritional ketosis, especially at the upper range of euketonemia, may be necessary to achieve optimal therapeutic outcomes, but this hypothesis needs to be rigorously tested. Further research is needed to fully elucidate chronic physiological adaptations and refine disease-specific protocols. Future efforts should prioritize optimizing personalized regimens that balance mechanistic insights with practical clinical translation, ensuring interventions align with both pathological targets and patient-specific needs.

## Key References

- 90. Gopalasingam N, Moeslund N, Christensen KH, Berg‐Hansen K, Seefeldt J, Homilius C, et al. Enantiomer‐Specific Cardiovascular Effects of the Ketone Body 3‐Hydroxybutyrate. Journal of the American Heart Association. 2024;13:e033628.
  - One of the few published papers designed to assess the effects of S-/L-BHB. Authors infused pigs in a randomized crossover with ketones vs an isovolumic control to assess cardiac function and health using catheterization and positron emission tomography imaging (PET).
- 123. Berg-Hansen K, Gopalasingam N, Christensen KH, Ladefoged B, Andersen MJ, Poulsen SH, et al. Cardiovascular Effects of Oral Ketone Ester Treatment in Patients With Heart Failure With Reduced Ejection Fraction: A Randomized, Controlled, Double-Blind Trial. Circulation. 2024;149:1474–89.
  - First published paper to investigate sustained (14 day) ketone ester intervention compared to a placebo, in a cardiovascular disease population. Investigators used imaging techniques and right heart catheterization to characterize cardiac function and pressures at rest and during exercise.

## Data Availability

No datasets were generated or analysed during the current study.
